# Ultrathin metal-organic framework array for efficient electrocatalytic water splitting

**DOI:** 10.1038/ncomms15341

**Published:** 2017-06-05

**Authors:** Jingjing Duan, Sheng Chen, Chuan Zhao

**Affiliations:** 1 School of Chemistry, Faculty of Science, The University of New South Wales, Sydney, New South Wales 2052, Australia

## Abstract

Two-dimensional metal-organic frameworks represent a family of materials with attractive chemical and structural properties, which are usually prepared in the form of bulk powders. Here we show a generic approach to fabricate ultrathin nanosheet array of metal-organic frameworks on different substrates through a dissolution–crystallization mechanism. These materials exhibit intriguing properties for electrocatalysis including highly exposed active molecular metal sites owning to ultra-small thickness of nanosheets, improved electrical conductivity and a combination of hierarchical porosity. We fabricate a nickel-iron-based metal-organic framework array, which demonstrates superior electrocatalytic performance towards oxygen evolution reaction with a small overpotential of 240 mV at 10 mA cm^−2^, and robust operation for 20,000 s with no detectable activity decay. Remarkably, the turnover frequency of the electrode is 3.8 s^−1^ at an overpotential of 400 mV. We further demonstrate the promise of these electrodes for other important catalytic reactions including hydrogen evolution reaction and overall water splitting.

## Abstract (web summary)

Metal-organic frameworks are generally considered to be inert catalysts for many electrochemical reactions, however this is not always the case. Here the authors fabricate an ultrathin nanosheet array of metal-organic frameworks exhibiting enhanced performance toward electrocatalytic water splitting.

Metal-organic frameworks (MOFs), constructed by joining metal ions with organic ligands, have emerged as a class of versatile porous materials for a wide range of applications^1^,^2^. However, MOFs are generally considered to be poor electrocatalysts for electrochemical reactions such as the oxygen evolution reaction (OER) and hydrogen evolution reaction (HER), the two core processes for electrochemical water splitting^3^,^4^,^5^. Taking OER as an example, the state-of-the-art MOFs operate at a energy cost significantly above thermodynamic requirements, showing a high overpotential and small turnover frequency (TOF) during oxygen evolution^3^.

The activity of an electrocatalyst is usually dependent on, among many other factors, accessible active centres, electrical conductivity and electrode geometry. Improvement in catalytic efficiency requires each of these parameters to be optimized, but increasing one of them without compromising the others is difficult. For example, MOFs have abundant intrinsic molecular metal sites, but few of them are utilized for electrocatalysis because of their poor electrical conductivity (usually ∼10^−10^ S m^−1^)^6^,^7^ and small pore size (usually within several nanometres)^1^. The recently reported strategies like calcinations at high temperature may sacrifice MOFs' intrinsic molecular metal active sites^8^, while hybridization with secondary conductive supports (polyaniline^9^, graphene^10^ and so on) may block their intrinsic micropores, and the bulk conductive MOF has limited meso- and macro-porosity (tens of nanometres to several micrometres) for effective mass transport during electrocatalysis^7^. Very recently, a few two-dimensional (2D) MOFs have been synthesized^11^,^12^,^13^,^14^,^15^, but the majority of 2D MOFs reported to date have been prepared in powder form, and little effort has been devoted to increasing the macro-/meso-porosity, conductivity or number of catalytic centres.

In this work, we develop a strategy for the *in situ* growth of ultrathin nanosheet arrays of 2D MOFs on various supports. Unexpectedly, the integrated MOF electrodes demonstrate superior performances towards OER, HER and overall water splitting. The material is prepared via a facile one-step chemical bath deposition method (Fig. 1) by adding the organic ligand (2,6-naphthalenedicarboxylic acid dipotassium) into an aqueous solution of metal salt (nickel acetate and iron nitrate) in the presence of the substrate. As shown in the right corner of Fig. 1, the crystal structure of the MOF consists of alternating organic hydrocarbon (2,6-naphthalenedicarboxylic group) and inorganic metal-oxygen-layers (MO_6_ units; M=Ni, Fe or Cu). The metal ions (Ni, Fe or Cu) are octahedrally coordinated, and each metal ion is coordinated to two trans monodentate carboxylates and four equatorial water molecules, while each naphthalene dicarboxylate bridges two metal atoms^16^,^17^. Moreover, the controlled experiments show that the 2D sheet-array morphology of the MOF cannot be obtained without using metal salts (Supplementary Fig. 1), substrates (Supplementary Fig. 2) or in different addition order of precursors (Supplementary Fig. 3). We further investigate the growth mechanisms by examining samples obtained at different reaction durations. NiFe-MOF evolves from micro-rods at 3 h (Supplementary Fig. 4a,b) into small-size nanosheets at 10 h (Supplementary Fig. 4c,d), and then large-size nanosheets at 20 h (Fig. 2), which is very similar to the previous reports of a dissolution-crystallization mechanism for nanocrystal growth^18^.

## Results

### Characterizations of the NiFe-MOF electrode

**Figure 2:**
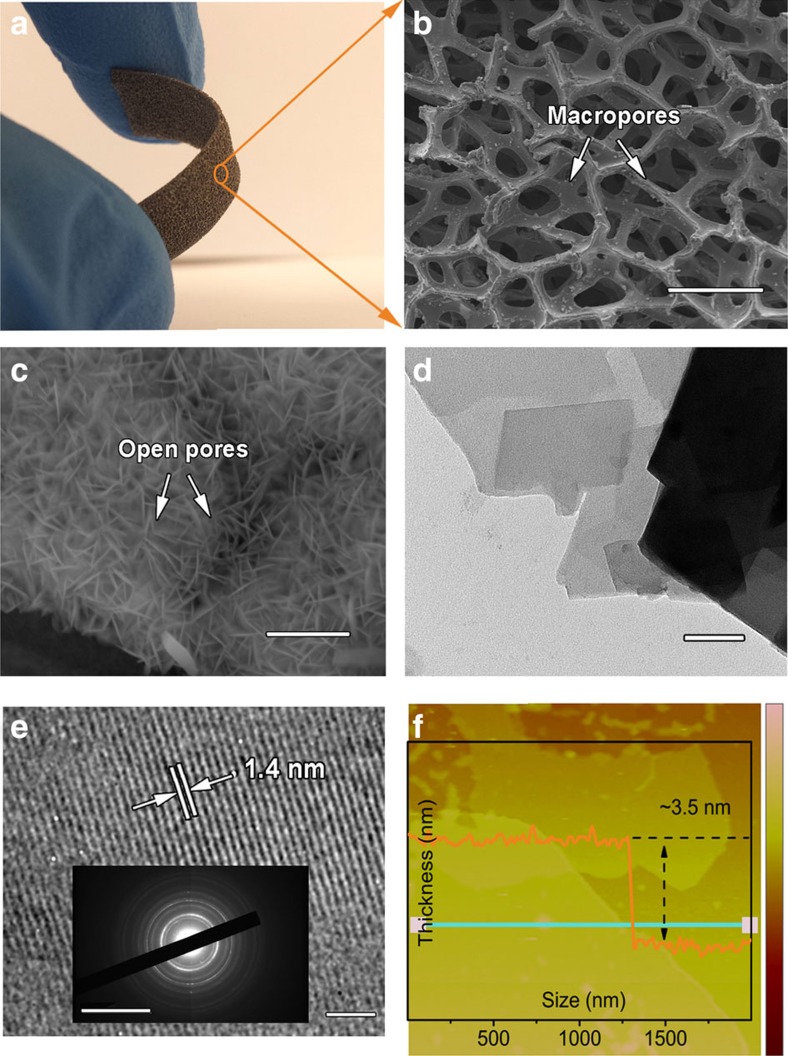
Morphological characterization of NiFe-MOF electrodes. (**a**) An optical image (size: 1 cm × 3 cm × 1 mm). (**b**,**c**) SEM images (scale bars are 300 μm for **b** and 1 μm for **c**). (**d**) Transmission electron microscopy image (Scale bar, 100 nm). (**e**) High-resolution transmission electron microscopy image (Scale bar, 5 nm) and selected area electron diffraction (SAED) pattern (scale bar, 10/1 nm). (**f**) AFM image and corresponding height profile along the marked green line.

The structure and morphology of NiFe-MOF was characterized with a number of techniques. The photograph in Fig. 2a shows that the NiFe-MOF grown on nickel foam (NiFe-MOF/NF) material is a 3D macroscopic film with high flexibility, which can be easily made into different sizes. The material is rich in macroporosity, with the pore size between 200 and 400 μm (Fig. 2b). The surface of the film is composed of an array of vertically grown nanosheets with the distance between adjacent layers around tens of nanometres (Fig. 2c). The nanosheets have a lateral size of several hundred nanometres (Fig. 2d) with smooth well-defined morphology. The pores inside the NiFe-MOF range from 2.5 to 18 nm as determined by nitrogen adsorption (Supplementary Fig. 5)^19^. Despite their rich porosity, these MOF nanosheets have a well-defined crystalline structure as revealed by the clear lattice fringes (∼1.4 nm lattice spacing due to the slit-like pores formed between adjacent metal-organic carbon layers, Fig. 2e), concentric circular rings of SAED pattern (inset of Fig. 2e), as well as X-ray diffraction (Supplementary Fig. 6). The thickness of nanolayers is determined to be ∼3.5 nm by atomic force microscopy (AFM) (Fig. 2f).

Moreover, X-ray photoemission spectroscopy (XPS) analysis reveals that the material contains Ni, Fe, O and C as the main components (Supplementary Fig. 7), where the Fe/Ni atomic ratio is 23%; therefore we determine the chemical formula of NiFe MOF as Ni_0.8_Fe_0.2_(C_12_H_6_O_4_)(H_2_O)_4_. This result is comparable to the elemental analysis conducted using both energy-dispersive X-ray spectroscopy and inductively coupled plasma with optical emission spectrometer (ICP-OES, please see Supplementary Fig. 8). Moreover, the C1s XPS spectrum shows the presence of both α,β-carbon and π-π* carbon that originated from the naphthalene ring of the organic ligand. The XPS Ni_2p3/2_ peak located at 856 eV indicates that Ni has an oxidation state of +2 in the MOF structure. Further, XPS Fe_2p1/2_ at 723 eV indicates that Fe has an oxidation state of +3 inside the material.

### Electrocatalytic oxygen and hydrogen evolution

**Figure 3:**
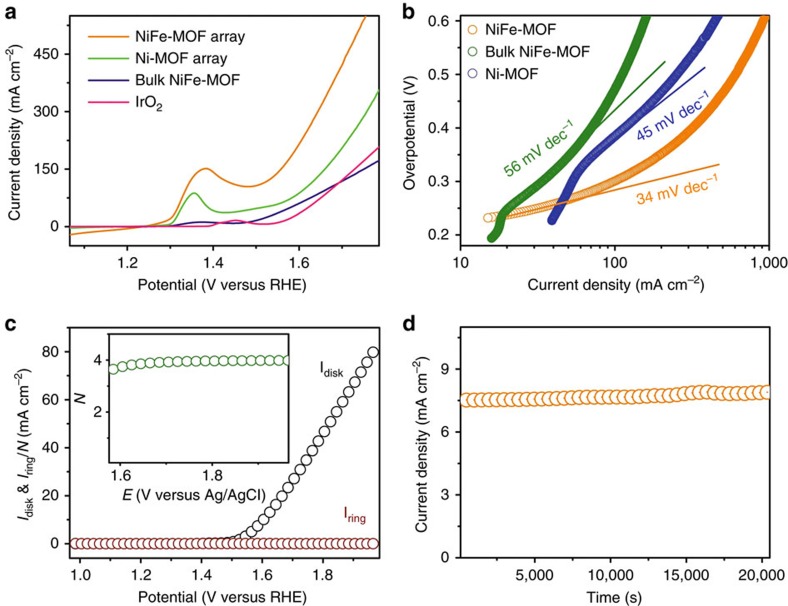
Electrocatalytic properties of NiFe-MOF and other samples for OER. (**a**) LSV plots obtained with NiFe-MOF, nickel-based metal-organic framework (Ni-MOF), bulk NiFe-MOF and IrO_2_ for OER at 10 mV s^−1^ in 0.1 M KOH. (**b**) Tafel plots obtained with NiFe-MOF, Ni MOF and bulk NiFe-MOF. (**c**) Rotate ring disk electrode voltammogram obtained for NiFe-MOF in 0.1 M KOH. The ring potential is set at 1.4 V versus reversible hydrogen electrode (RHE) to monitor the production of hydrogen peroxide; the inset of (**c**) shows the corresponding electron transfer number (*N*) as a function of applied potentials. (**d**) Chronoamperometric testing of NiFe-MOF for 20,000 s at 1.42 V (versus RHE) in 0.1 M KOH.

The electrocatalytic performance of NiFe-MOF for OER (the anodic reaction of water splitting) was tested in 0.1 M KOH electrolyte in a typical three-electrode system. All the data were acquired without *iR*-correction. According to the linear sweep voltammograms (LSV) of Fig. 3a and Supplementary Figs 9 and 10 (The reverse scan of LSVs) the NiFe-MOF demonstrated an overpotential of 240 mV at a current density of 10 mA cm^−2^, which is much smaller than other controlled samples including the Ni-MOF (without Fe, 296 mV), Fe-MOF/NF (without Ni, 354 mV), nickel foam (without MOF, 370 mV), NiFe-MOF powder loaded on NF (denoted as bulk NiFe-MOF, without mesopores, 318 mV), calcined NiFe-MOF (at 650 °C for 6 h in N_2_, without molecular NiFe sites, 336 mV) and NiFe-MOF grown on a glassy carbon macrodisc electrode (NiFe-MOF/GC, without macro- and meso-porosity, 406 mV). Even compared with the commercial benchmark OER catalyst IrO_2_, NiFe-MOF shows a significantly smaller overpotential @10 mA cm^−2^ (240 versus 320 mV) and 2.7 times higher current density at 1.7 V versus RHE (300 versus 80 mA cm^−2^). Moreover, assuming all the Ni and Fe sites inside NiFe-MOF are involved in OER, the TOF of 3.8 s^−1^ is obtained for NiFe-MOF at an overpotential of 400 mV. In contrast, the TOF of benchmark IrO_2_ is only 0.14 s^−1^. Supplementary Table 1 also summarizes a comparison of NiFe-MOF with recently reported OER electrocatalysts. It suggests that NiFe-MOF/NF is already one of the most efficient electrocatalysts for OER reported in the literature, to the best of our knowledge.

The superior OER performance of NiFe-MOF electrode was also confirmed by its smaller Tafel plots derived from LSVs (34 mV dec^−1^, Fig. 3b and Supplementary Fig. 11) than other controlled samples such as Ni-MOF (45 mV dec^−1^), bulk NiFe-MOF (56 mV dec^−1^) and IrO_2_ (43 mV dec^−1^). This result is consistent with that obtained from the steady state test (38 mV dec^−1^ for NiFe-MOF and 46 mV dec^−1^ for IrO_2_; Supplementary Fig. 12). The average electron transfer number (N) of NiFe-MOF obtained by using a rotating ring-disk electrode is 3.95 (Fig. 3c and Supplementary Figs 13,14); the reaction Faradic efficiency was 95±2.5%, both indicating OER follows a four-electron pathway (that is, 4OH^−^→2H_2_O+O_2_+4e^−^) to generate oxygen molecules. Remarkably, the NiFe-MOF electrode showed excellent stability as confirmed by prolonged chronoamperometric experiment (fixed at 1.42 V versus RHE for 20,000 s, Fig. 3d), cyclic voltammetry (1,000 cycles, Supplementary Fig. 15a) and electrochemical impedance spectroscopy (EIS, Supplementary Fig. 15b).

**Figure 4:**
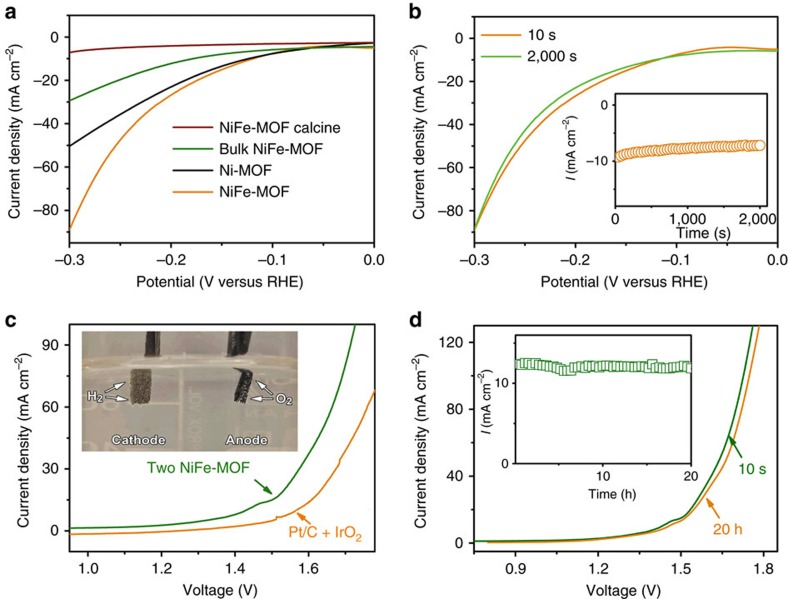
Electrocatalytic properties of NiFe-MOF and other samples for HER and overall water splitting. (**a**) LSV plots obtained with NiFe-MOF, bulk NiFe-MOF, Ni-MOF and calcined NiFe-MOF for HER at 10 mV^−1^ in 0.1 M KOH. (**b**) LSV of NiFe-MOF for HER before and after chronoamperometric testing for 2000, s at −0.2 V (versus RHE) in 0.1 M KOH; the inset of (**b**) shows corresponding chronoamperometric profile. (**c**) LSV plots of a full electrolytic cell using two NiFe-MOF electrodes obtained at 10 mV s^−1^ in 0.1 M KOH; the inset photograph shows the evolution of hydrogen and oxygen gas bubbles at the NiFe-MOF electrodes at an applied cell voltage of 1.6 V; the LSV obtained using a Pt/C cathode and a IrO_2_ anode was included for comparison. (**d**) LSV plots of the two-electrode electrolytic cell obtained before and after 20 h chronoamperometric tests at a cell voltage of 1.5 V; the inset shows corresponding chronoamperometric plot.

Furthermore, the NiFe-MOF is also highly active towards HER, the cathodic reaction of water splitting. The electrocatalytic performance of NiFe-MOF for HER was tested in 0.1 M KOH by polarizing the electrode to negative potentials (Fig. 4a). At the current density of 10 mA cm^−2^, NiFe-MOF exhibits the smallest overpotential of 134 mV, compared to other samples including Ni-MOF (177 mV), bulk NiFe-MOF (196 mV) and calcined NiFe-MOF (255 mV). The TOF of NiFe-MOF for HER at the overpotential of 400 mV is 2.8 s^−1^, which outperforms that of Ni-MOF (0.91 s^−1^), bulk NiFe-MOF (0.53 s^−1^) and calcined NiFe-MOF (0.19 s^−1^). This performance is comparable to state-of-the-art non-precious metal based HER catalysts (Supplementary Table 2). Further, prolonged chronoamperometric experiment at −0.2 V (versus RHE, Fig. 4b) for 2,000 s showed a stable current response, and the LSVs obtained before and after chronoamperometric test are also identical, all indicating excellent durability of the NiFe-MOF for HER.

### Electrocatalytic overall water splitting

To test the overall water splitting, a two-electrode cell was constructed by using NiFe-MOF as both the anode and the cathode. The photograph (inset of Fig. 4c) and corresponding movie (Supplementary Movie 1) reveal that at the applied cell voltage of 1.6 V, a large amount of H_2_ gas bubbles evolve at the cathode and O_2_ gas bubbles evolve at the anode, also confirmed by gas chromatography (Supplementary Fig. 16). The electrolytic cell demonstrated excellent catalytic activity and can deliver a current density of 10 mA cm^−2^ at a voltage only of 1.55 V (Fig. 4c), which is 70 mV smaller than using the benchmark precious metal-based Pt/C cathodes and IrO_2_ anodes. The Tafel slope obtained for NiFe-MOF (256 mV dec^−1^) is also lower than the Pt/C cathode and IrO_2_ anode system (267 mV dec^−1^, Supplementary Fig. 17). To the best of our knowledge, the NiFe-MOF outperforms most, if not all, bifunctional electrocatalysts reported for full water splitting^20–22^. Furthermore, the electrolytic cell demonstrates excellent stability in a prolonged chronoamperometric test at 1.5 V for 20 h (inset of Fig. 4d). The durability of water electrolysis is further supported by the almost identical LSVs (Fig. 4d), X-ray diffraction profiles (Supplementary Fig. 18), and scanning electron microscopy (SEM) images (Supplementary Fig. 19) obtained before and after the chronoamperometric test.

## Discussion

The materials' mechanisms for different reactions have been discussed. First, it is generally accepted that Ni-based catalysts have three intermediate steps in the OER^22^,^23^ (E^0^ versus RHE, reversible hydrogen electrode), that is:


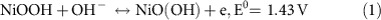



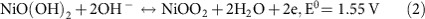



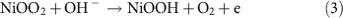



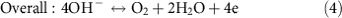


Step (1) and (2) are highly reversible, while step (3) is fast and irreversible and determines the overall rate of the process; catalysts are mostly used to facilitate the kinetics of step (3). In the anodic OER process, NiO_6_ inside MOF was oxidized into NiO_6_/NiOOH species as active centres, which then promote the oxidation of OH^−^ into molecular oxygen. In addition, 23 wt% of Fe impurity enhances the activity of Ni-based catalysts through introducing additional structural vacancies (Supplementary Fig. 20)^23^,^24^.

Second, the HER pathway in alkaline media could be the Volmer–Heyrovsky process or Volmer–Tafel pathways:


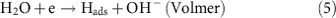


and


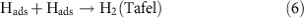


or


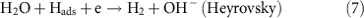


Both pathways involve the adsorption of H_2_O, electrochemical reduction into adsorbed H atom and OH^−^, and desorption of H_2_. In this work, NiO_6_ inside MOF was partially reduced to form Ni/NiO_6_ interface at the cathode. On such interface, the OH^−^ generated by H_2_O splitting could preferentially attach to a NiO_6_ site at the interface due to strong electrostatic affinity to the locally positively charged Ni^2+^ species, while a nearby Ni site would facilitate H adsorption and thus the Volmer process, imparting synergistic HER catalytic activity^25^.

The significantly enhanced performance of NiFe-MOF catalyst for water splitting has been further discussed, which is attributable to its optimal structural characteristics for electrocatalysis including highly exposed molecular metal active sites owing to ultrathin MOF sheets, improved electrical conductivity through 2D nanostructuration, and a combination of hierarchical porosity. First, NiFe metal oxides/hydroxides are active catalytic centres for OER, HER and overall water splitting; however, they usually show compromised catalytic performance owing to limited exposed metal active sites^22^. In contrast, MOF material has inherent molecular metal centres, as potential active sites for electrocatalysis. The direct use of MOF as electrocatalyst can provide enormous molecular metal sites as catalytic centres; while the ultrathin (thickness, ∼3.5 nm) yet large (lateral size >100 nm) 2D MOF nanosheets structure allows for these metal sites to be highly exposed to electrolyte ions for use in catalytic reactions. This is confirmed by a two-fold increase of electrochemical active surface measured by using double-layer capacitance of the 2D NiFe-MOF nanosheets, compared with the bulk NiFe-MOF (0.036 versus 0.016 F cm^−2^, Supplementary Fig. 21). Interestingly, the highly exposed metal NiFe centres also render MOF nanosheets very hydrophilic, as suggested by zeta potential (−29.2 mV, Supplementary Fig. 22), contact angle (38^o^, Supplementary Fig. 23) and Fourier transform infrared (FT-IR) spectra analyses (Supplementary Fig. 24), which can facilitate the adsorption of water onto electrode surface, and promote the kinetics of water dissociation^26^.

Moreover, we scratched 2D MOF nanosheets from nickel foam substrates, and then pressed it at 10 MPa for 3 min to form a uniform thin film of thickness ∼240 nm (Supplementary Fig. 25). We found that the 2D MOF nanosheets have intrinsic high electrical conductivity (1±0.2 × 10^−3^ S m^−1^) measured by a four-point probe, which is almost three orders of magnitude higher than its bulk counterpart (1±0.5 × 10^−6^ S m^−1^). Such enhancement is generally due to 2D nanostructuration that can lead to vacancy engineering inside the material. These vacancies can act as shallow donors to increase the carrier concentration of metal octahedral units (that is, NiFeO_6_) of the MOF, thereby enhancing the conductivity of materials^27^,^28^,^29^. The high conductivity can facilitate the charge transport during electrocatalysis, thus contributing to the observed high activity of the 2D NiFe-MOF. Moreover, the direct growth of NiFe-MOF on nickel foam can eliminate the need for insulating chemical binders. Generally, to construct powders of MOFs into usable electrodes, insulating polymeric binders, for example Nafion, are required to glue these materials to a substrate electrode, which can reduce the contact area between electrolytes and catalytic active sites and deteriorate the overall conductivity of electrode. This is confirmed by the large internal resistance of bulk NiFe-MOF/NF (prepared by the Nafion-assisted drop-casting method, 8.2 Ω) than NiFe-MOF (2.8 Ω, prepared by the direct growth method, Supplementary Fig. 26). The enhanced catalyst-substrate contact on binder-free electrode contribute to efficient electron transport and consequently high catalytic activity.

Further, the unique hierarchical porous architecture of NiFe-MOF sheet array on nickel foam also contributes to the enhanced activity by ensuring effective mass transport within the electrodes (Fig. 3a–c)^30^. One of the major challenges of using MOFs for electrocatalysis is the very small pore size (usually within several nanometres) of bulk MOF materials, which inhibits the effective mass transport of electrolyte to the active centres and diffusion of products, leading to impeded electrode performance. This is particularly a problem in water splitting as the electrode products are O_2_ and H_2_ gases, which can potentially block the active sites of MOFs and prohibit ionic transportation, and is one of the major sources of overpotential. Here, the as-prepared NiFe-MOF electrode demonstrates a combination of hierarchical-scale porosity (several-hundred-micrometre macropores, tens-of-nanometre open pores, several-nanometre mesopores and intrinsic microporosity). The extra-large macropores of nickel foam can facilitate the mass transport of electrolytes and gaseous products (Fig. 2a,b), while the open pores between vertically aligned nanosheets (Fig. 2c), small mesopores and micropores of MOF (Fig. 2e and Supplementary Fig. 5) provide enormous, highly accessible active sites and short ion diffusion pathways.

Finally, we show the synthetic approach is generic and can be adapted for a range of MOF materials and substrate materials. Supplementary Fig. 27 shows ultrathin NiFe-MOF nanosheet can be grown on stainless steel mesh via a similar procedure. SEM images in Supplementary Fig. 27 show the stainless steel mesh is coated by numerous ultrathin nanosheets of NiFe-MOF with the lateral size of several micrometres and thickness of a few nanometres. Supplementary Fig. 28 shows the synthetic procedure is also adaptable to other transition metals-based MOF materials, such as copper-based MOF (denoted as Cu-MOF) on nickel foam. AFM reveals the nanosheets have typical thickness of 6.8 nm and lateral size of tens of nanometres (Supplementary Fig. 27c,d). All the above data suggest the 2D MOF nanosheet array is a promising class of materials for electrochemical applications.

In summary, this work demonstrates a universal strategy to fabricate ultrathin nanosheet arrays of 2D MOFs, which can be easily adaptable to prepare many other metal-based 2D MOFs such as cobalt, manganese, titanium and molybdenum. The as-resultant material combines a number of remarkable features, and exhibited significantly enhanced catalytic performances with high catalytic activity, favourable kinetics and strong durability towards electrocatalysis such as OER, HER and overall water splitting. The performance of our electrode for water splitting challenges a common conception that MOFs themselves are an inert catalyst for electrochemical reactions.

## Methods

### Synthesis of NiFe-MOF electrode

A piece of nickel foam (NF) (2 cm × 1 cm × 1.6 mm) or stainless steel mesh, was immersed into a vial containing 1 ml of DI-water and 8 mg of Ni(Ac)_2_·4H_2_O and 2 mg of Fe(NO_3_)_3_·9H_2_O. Next, 10 mg of organic ligand 2,6-naphthalenedicarboxylate tetrahydrate was added into the above solution, and the vial was sealed for reaction at 60 °C for 20 h. After cooling down to room temperature, the nickel foam was taken out, bath ultrasonicated for 1 min and then rinsed with copious DI-water.

### Physical characterizations

X-ray diffractionwas performed on a Philips 1130 X-ray diffractometer (40 kV, 25 mA, Cu Kα radiation, *λ*=1.5418 Å); the contact angle was measured on a Theta/Attension Optical Tensiometer; the electrical conductivity was measured on a Signatone Four Point Probing System with the MOF nanosheets scratched out and pressed into a circular pellet (1 cm in diameter and 43 μm thick); XPS was performed on an Axis Ultra (KratosAnalytical, UK) XPS spectrometer equipped with an Al Ka source (1486.6 eV); inductively coupled plasma optical emission spectrometer (ICP-OES) methodology was used for elemental analysis conducted on a Thermo Scientific iCAP 6500 duo optical emission spectrometer fitted with a simultaneous charge induction detector; FT-IR spectra were recorded on a Nicolet 6700 spectrometer; zeta potential was monitored on a Malvern Zetasizer Nano series analyser; GC was conducted on the Shimadzu GC-2010; AFM was conducted on Bruker Dimension ICON SPM using peak force mode; morphologies of the samples were observed on transmission electron microscopy (TecnaiG2Spirit and JEOL JEM-ARM200F) and SEM (QUANTA 450); energy-dispersive X-ray spectroscopy and element mapping were acquired on the SEM (QUANTA 450). Further, the porosity was evaluated by using nitrogen adsorption–desorption isotherms measured at 77 K on a TriStar II 3020 Micrometrics apparatus.

### Electrochemical characterizations

OER and HER were studied in a standard three-electrode glass cell connected to a 760 workstation (Pine Research Instruments, US) using the NiFe-MOF as the working electrode, carbon rod as a counter electrode and Ag/AgCl/KCl (3 M) as a reference electrode. All the measured potentials were converted to reversible hydrogen electrodes (RHE) according to Potential=E_Ag/AgCl_+0.059 pH+0.197 V. The two-electrode system was built by employing two NiFe-MOF electrodes. The electrolyte was prepared using DI-water (18 MΩ cm^−1^) and KOH. LSV and CV were recorded with the scan rates of 10 mV s^−1^; Tafel plots are recorded with the linear portions at low overpotential fitted to the Tafel equation (*η*=*b* log *j*+*a*, where *η* is overpotential, *j* is the current density, and *b* is the Tafel slope; EIS was recorded under the following conditions: AC voltage amplitude 0 or 1.5 V, frequency ranges 10^6^ to 1 Hz, and open circuit; the current density was normalized to the geometrical area; All the electrochemical data were presented without *iR* correction.

### Data availability

The data that support the plots within this paper and other findings of this study are available from the corresponding author on request.

## Additional information

**How to cite this article:** Duan, J. *et al*. Ultrathin metal-organic framework array for efficient electrocatalytic water splitting. *Nat. Commun.* **8,** 15341 doi: 10.1038/ncomms15341 (2017).

**Publisher's note:** Springer Nature remains neutral with regard to jurisdictional claims in published maps and institutional affiliations.

## Supplementary Material

Supplementary Information Supplementary Figures, Supplementary Tables, Supplementary Note, Supplementary Methods and Supplementary References

Supplementary Movie 1 NiFe-MOF used as both the cathode and anode for water splitting at the voltage of 1.2, 1.4, 1.6, and 1.8 V in 0.1 M KOH electrolyte. The video shows that hydrogen bubbles evolve from the cathode, while oxygen bubble evolves from the anode. Even at high operating voltage of 1.8 V, no significant catalyst peeling off is observed, suggesting the strong adhesion of NiFe-MOF on nickel foam substrate.

## Figures and Tables

**Figure 1:**
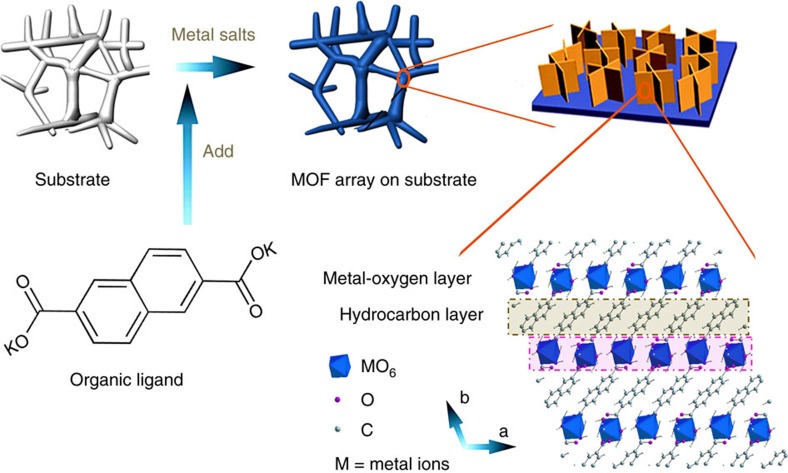
Synthetic process of metal-organic framework nanosheet array. Metal salts and substrate are firstly mixed together in an aqueous solution, and then introduces the organic ligand. Next, the MOF nanosheet array grows on the surface of substrate via a dissolution-crystallization mechanism.
