# Supplementary material for: Ultrathin metal-organic framework array for efficient electrocatalytic water splitting
Source: Nat Commun. 2017 Jun 5;8:15341. doi: 10.1038/ncomms15341 (PMC5465318; doi:10.1038/ncomms15341)
Supplement: Supplementary Information — Supplementary Figures, Supplementary Tables, Supplementary Note, Supplementary Methods and Supplementary References [file ncomms15341-s1.pdf]

Supplementary Methods

Chemicals. Nickel foam (NF, thickness 1.6 mm, bulk density 0.45 g cm^{-3}, porosity 95%), stainless steel mesh (300 mesh, bulk density 0.25 g cm^{-3}, wire diameter 0.04 mm, open area 28%), nickel acetate ($Ni(Ac)_2·4H_2O$, 98%), iron nitrate ($Fe(NO_3)_3·9H_2O$, 98%), copper(II) acetate ($Cu(Ac)_2$, 98%), 2,6-Naphthalenedicarboxylic acid dipotassium ($C_{10}H_6(CO_2K)_2$, 95%), iridium(IV) oxide (IrO_2, 99%), platinum carbon black (Pt/C, 20%), potassium hydroxide (KOH, 99%), were purchased from Sigma-Aldrich and directly used without further treatment or purification. All aqueous solutions were prepared with high-purity de-ionized water (DI-water, resistance 18 MΩ cm^{-1}).

Synthesis of bulk NiFe-MOF/NF. The synthesis of bulk material is similar to that of NiFe-MOF without using any substrates. The NiFe-MOF powder was dispersed in isoproponal/water (v/v = 1/3) mixed solvent with 1 wt% nafion as the binder, and then drop casted onto the NF substrate with a loading of 0.3 mg cm^{-2}

Calcination of NiFe-MOF. The NiFe-MOF was calcined at the temperature of 650 ^{o}C for 6 h with an elevated rate of 5 ^{o}C min^{-1} in N_2 atmospheres.

Synthesis of NiFe-MOF/stainless steel mesh. The sample was prepared by following a similar procedure to NiFe-MOF/NF except that stainless steel mesh was used as the support.

Synthesis of Cu-MOF/NF. The sample was prepared by following a similar procedure to NiFe-MOF on nickel foam except that 10 mg of $Cu(Ac)_2$ was used as the metal precursor.

Electrochemical tests. To calculate the Farady efficiency, the rotating ring-disk electrode (RRDE) voltammograms were conducted using a RRDE configuration (Pine Research Instrumentation, USA) comprised of a glassy carbon disk electrode and a

platinum ring electrode. The NiFe-MOF was scratched from nickel foam and then coated onto RRDE with Nafion as the binder (catalyst loading: 0.3 mg cm^{-2}). A scan rate of 10mV s^{-1} and a rotation rate of 1500 rpm were applied for LSV tests on RRDE. The ring potential of the RRDE was kept constantly at 0.4 V *vs.*RHE to reduce the O_2 produced from catalyst on the disk electrode in 0.1 M KOH solution. The electron transfer number (N) can be calculated from the disk current (I_d) and ring current (I_r) of the RRDE

$$N = 4\times I_d/ (I_d+ I_r/N)$$

Where I_d is disk current, I_r is ring current, and N is the current collection efficiency of RRDE, which was determined to be 0.37.

In addition, the turnover frequency (TOF) was calculated according to the following equation: TOF = j×A/(4×F×m),where j is the current density obtained at overpotential of 400 mV in A cm^{-2}, A is the surface area of the electrode, F is the Faraday efficiency (96,485 C mol^{-1}) and m is the number of moles of the Ni^{2+} and Fe^{3+}onthe electrodes

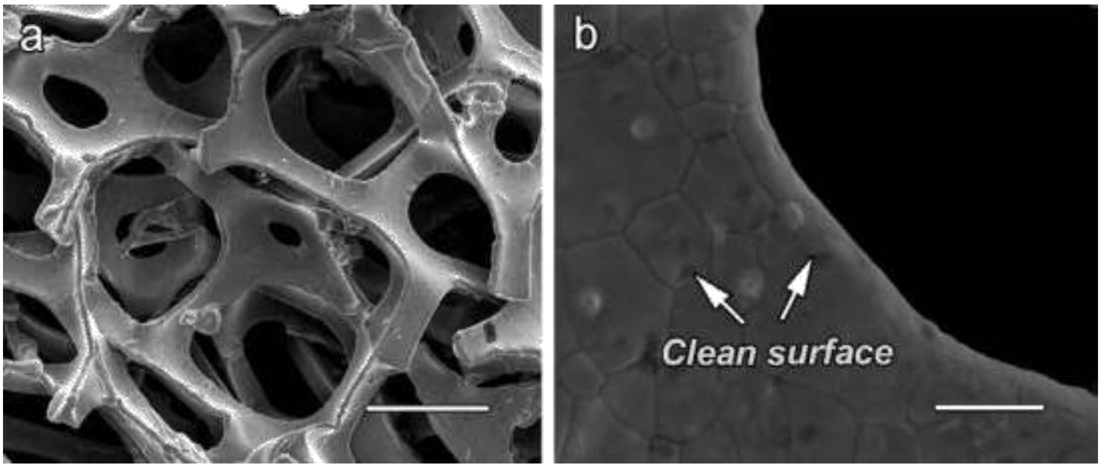

Supplementary Figure 1: SEM of the controlled sample prepared by using only organic ligand (2,6-Naphthalenedicarboxylic acid dipotassium) as precursor, showing that nickel foam has a clean surface which indicates the organic ligand does not deposit on nickel foam under the synthetic condition (scale bar for **a** represents for 200 μm, and for **b** for 10 μm).

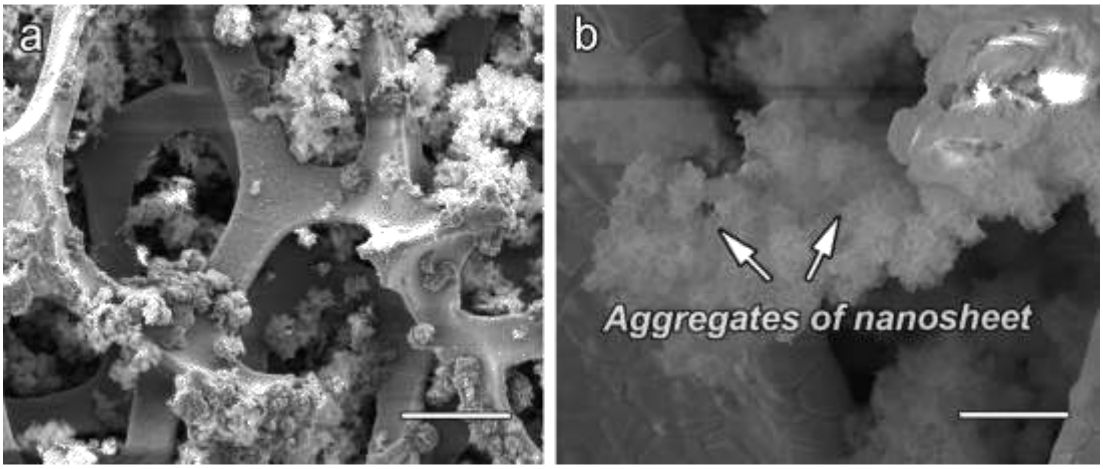

Supplementary Figure 2: SEM images of bulk NiFe MOF. The material is deposited onto nickel foam to make a better comparison to that of ultrathin nanosheet array. The bulk material is generally made of the aggregations of nanosheets, and has a typical secondary particle size of several micrometers (scale bar in **a** represents for 150 μm, and **b** for 10 μm).

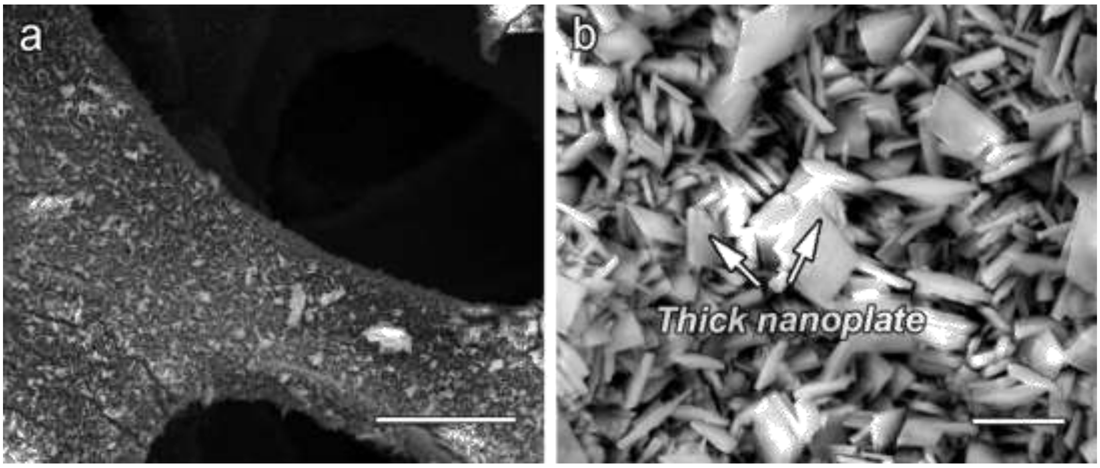

Supplementary Figure 3: SEM images of controlled sample prepared by firstly adding organic ligand (2,6-Naphthalenedicarboxylic acid dipotassium) and then nickel and iron salts, showing that thick nanoplate of NiFe MOF has formed during this process. A nanoplate has a typical lateral size of several micrometers and thickness of tens of nanometers, which is different from ultrathin thin nanosheet in the **Supplementary Figure 1** of main text (scale bar in **a** represents for 50 μm, and **b** for 5 μm).

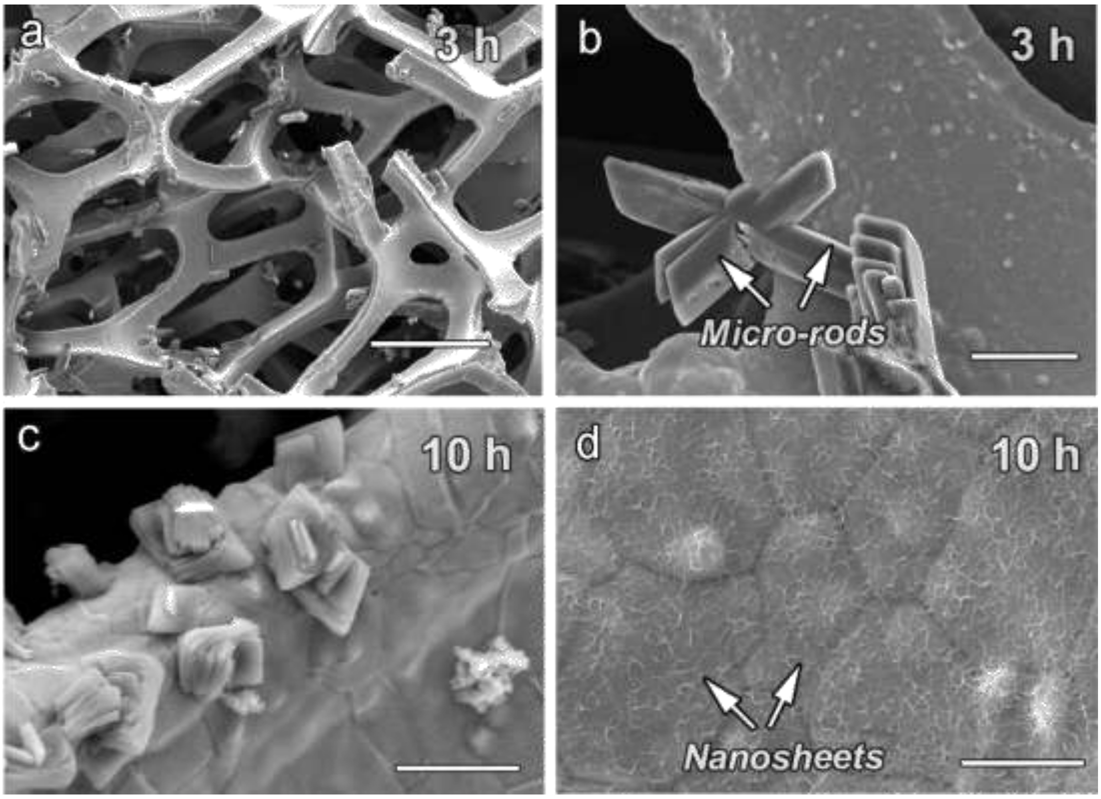

Supplementary Figure 4: The intermediate products of NiFe MOF at different synthetic durations: (**a**, **b**) at 3 h, where the metal salts and organic ligand forms large micro-rods of several micromters; (**c, d**) at 10 h the microrods have transfered into small nanosheets grown onto the surface of nickel foam. These data indicates a dissolution-crystallization mechanism for the formation of NiFe MOF ultrathin nanosheet (Chem. Mater. 2007, 19, 5410). The scale bars in **a-d** represent for 250, 20, 50, and 5 μm.

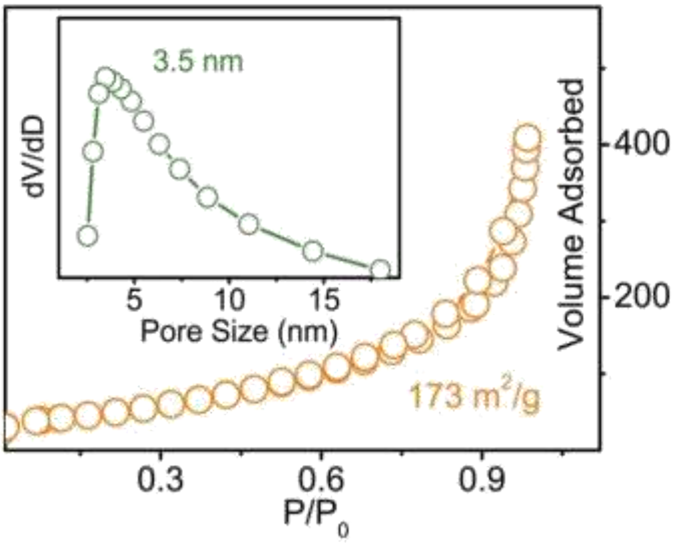

Supplementary Figure 5: Nitrogen adsorption-desorption isotherm of NiFe-MOF.

Supplementary Note 1: nitrogen adsorption was used to evaluate the porosity of NiFe-MOF; adsorption isotherm shown in the inset of **Supplementary Figure 5** resembles the type IV with a narrow hysteresis loop. The corresponding pore size distribution obtained by Barrett-Joyner-Halenda (BJH) method shows the presence of small mesopores ranging from 2.5 to 18 nm (centered at 3.5 nm, inset of **Supplementary Figure 5**). The Brunauer-Emmett-Teller (BET) surface area of NiFe-MOF was estimated to be 173 m^2 g^{-1}.

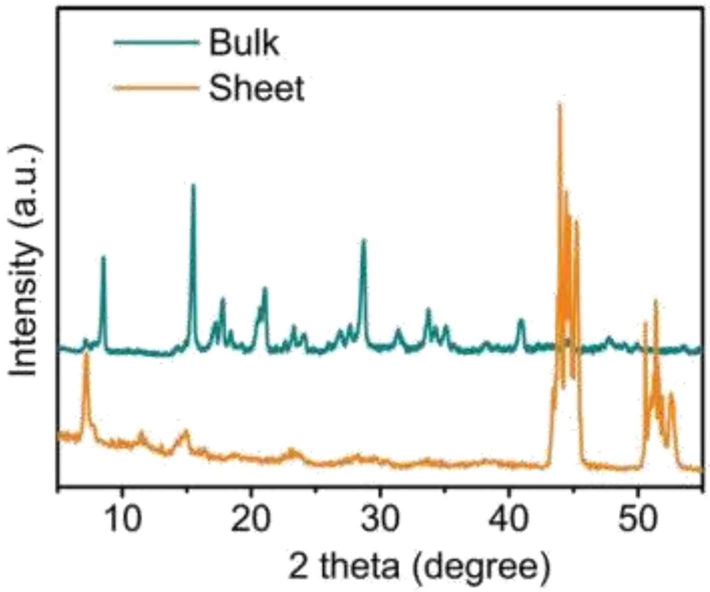

Supplementary Figure 6: XRD pattern in comparison to its bulk counterpart, which suggests the 2theta degrees of all the diffraction peaks are close to that of $Ni(C_{12}H_6O_4)(H_2O)_4$.

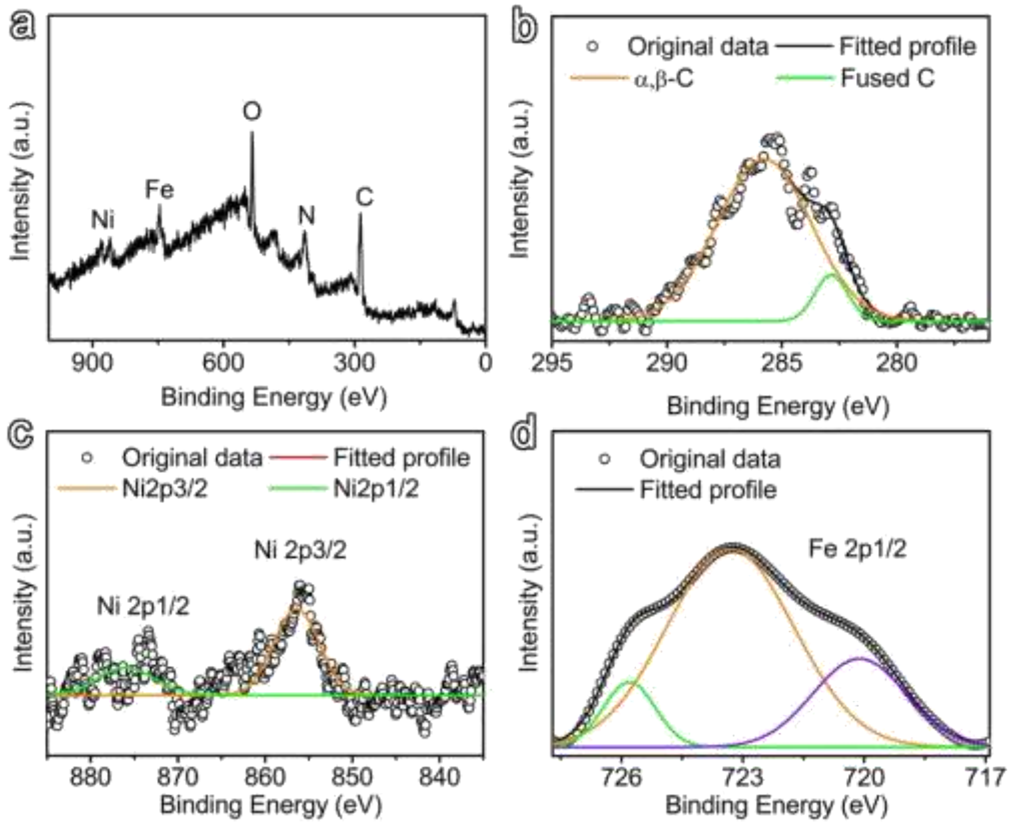

Supplementary Figure 7: XPS survey, C_{1s}, Ni_{2p} and Fe_{2p} spectra of NiFe-MOF.

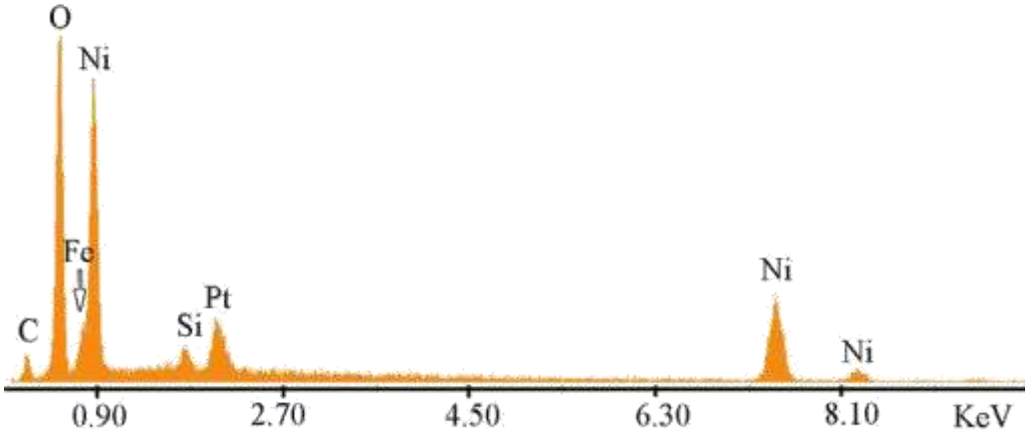

Supplementary Figure 8: Energy-dispersive X-ray spectroscopy of NiFe-MOF. The data reveals that the material contains Ni, Fe, O, and C as the main components. The Fe/Ni atomic ratio is 15%; therefore the chemical formula of NiFe-MOF is determined as $Ni_{0.86}Fe_{0.14}(C_{12}H_6O_4)(H_2O)_4$.

Supplementary Note 2: This result is consistent with that from Inductively Coupled Plasma with optical emission spectrometer (ICP-OES). The analysis shows that the mass concentrations of Ni and Fe in are 334 and 1849 mg L^{-1}, which corresponds to the Fe/Ni atomic ratio as 18%. Therefore the chemical formula of NiFe-MOF is calculated as $Ni_{0.83}Fe_{0.17}(C_{12}H_6O_4)(H_2O)_4$.

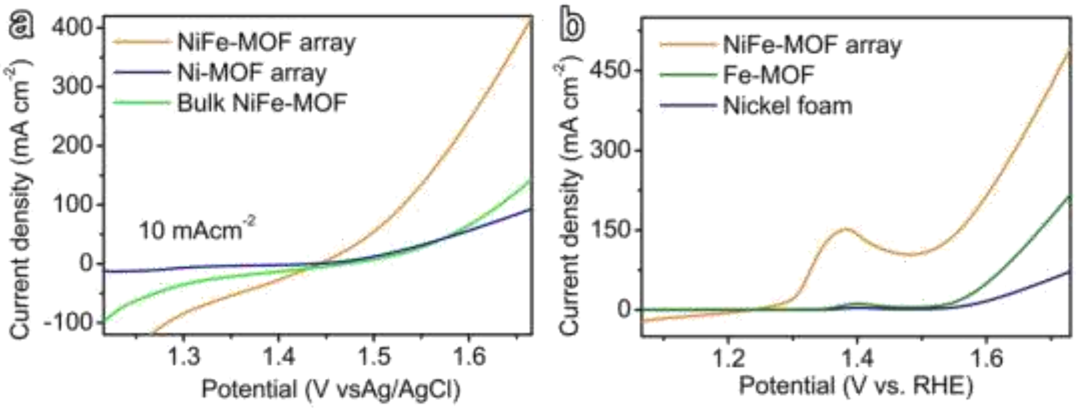

Supplementary Figure 9: LSVs of NiFe-MOF array in comparison to other samples. (**a**) The reverse scan from 1.66 to 1.22 V (*vs*. RHE) for NiFe MOF, Ni MOF, and bulk NiFe MOF at 10 mV^{-1} in 0.1 M KOH electrolyte; (**b**) LSV plots of NiFe-MOF in comparison to individual Fe-MOF and nickel foam.

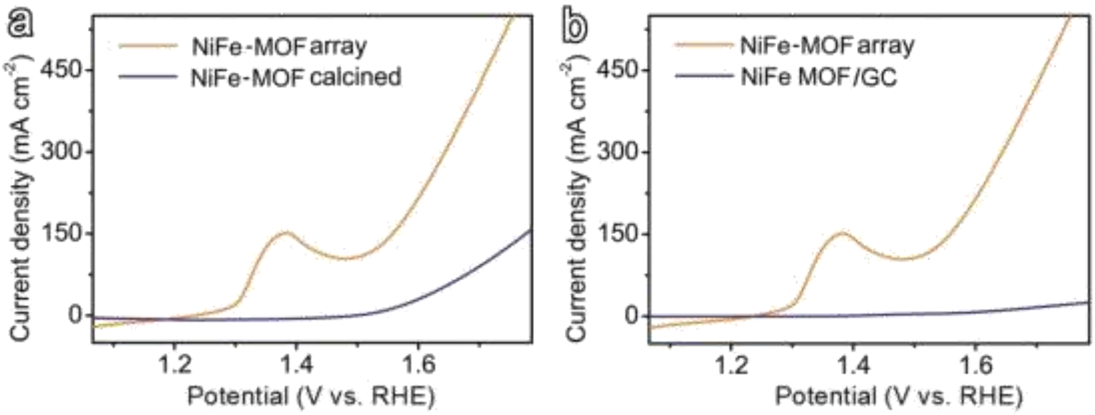

Supplementary Figure 10: LSV plots of NiFe-MOF in comparison to its calcined counterpart (**a**) and GC counterpart (**b**, prepared by drop casting of NiFe-MOF on glassy carbon with a mass loading of 0.3 mg cm^{-2}) at the scan rate of 10 mV s^{-1} in 0.1 M KOH electrolyte.

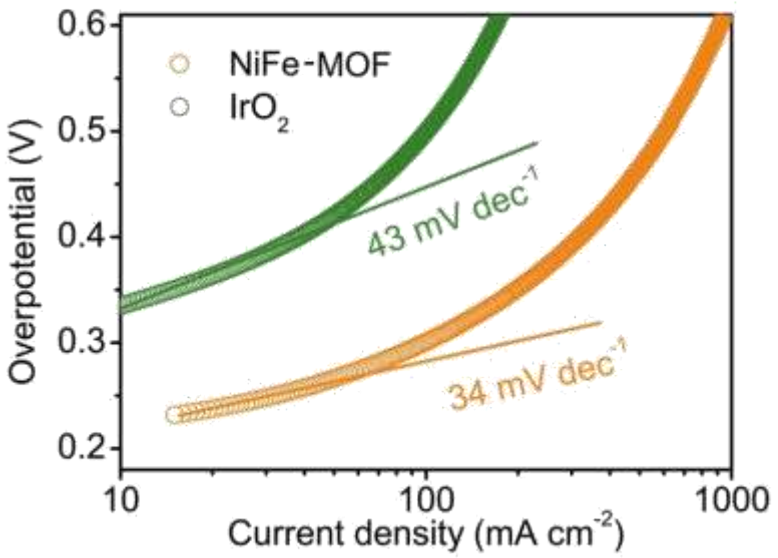

Supplementary Figure 11: Tafel plots of NiFe-MOF during OER process in comparison to IrO_2. The smaller Tafel slope of NiFe MOF (34 mV dec^{-1}) indicates its more favorable reaction kinetics than other samples.

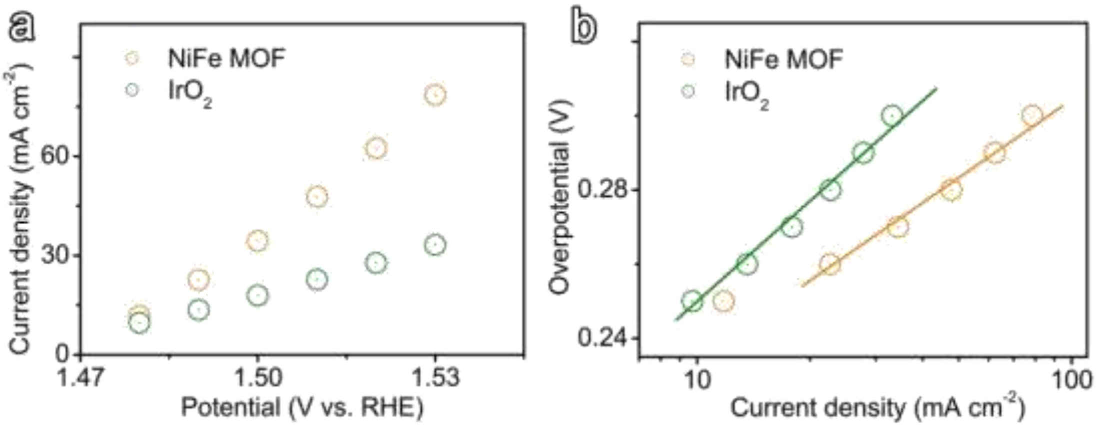

Supplementary Figure 12: Steady state test of NiFe-MOF and IrO_2. (**a**) Current-potential plot with each point recorded using amperometric i-t method after operation for 10 min; (**b**) corresponding Tafel plots.

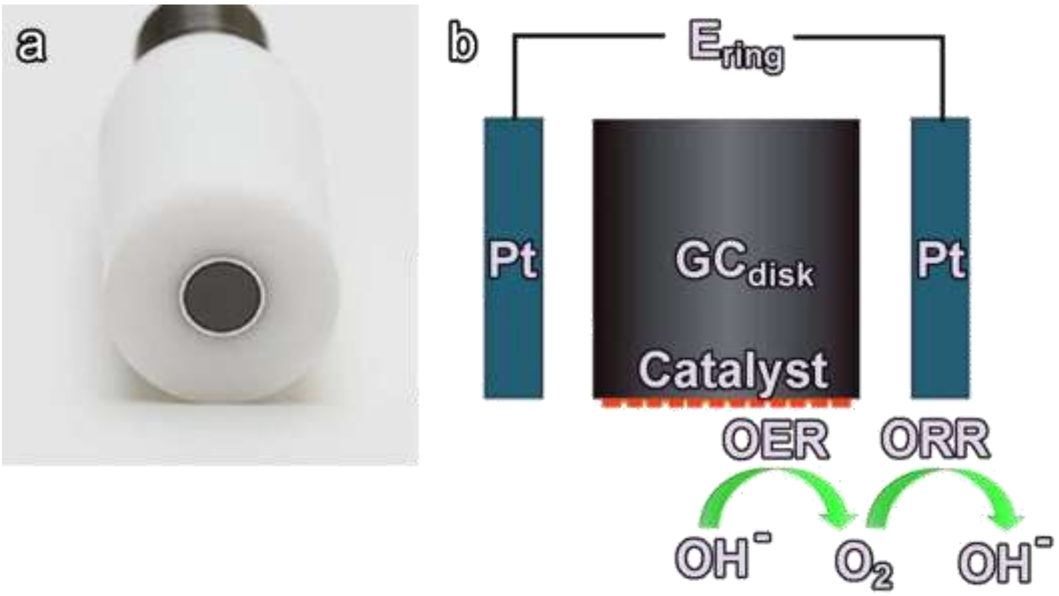

Supplementary Figure 13: Apparatus and mechanisms for RRDE testing. (**a**) the photograph of the used RRDE; (**b**) schematic illustration of using RRDE to detect the OER process during anodic polarization of NiFe-MOF in 0.1 M KOH solution. Water oxidation proceed on disk electrode will generate either O_2 (OER, $2H_2O \rightarrow 4H^+ + O_2 + 4e^-$), which will be subsequently reduced by Pt ring electrode. The continuous OER (GC disk electrode) → ORR (Pt ring electrode) process initiated on a RRDE will give rise to detectable current increase in ring electrode.

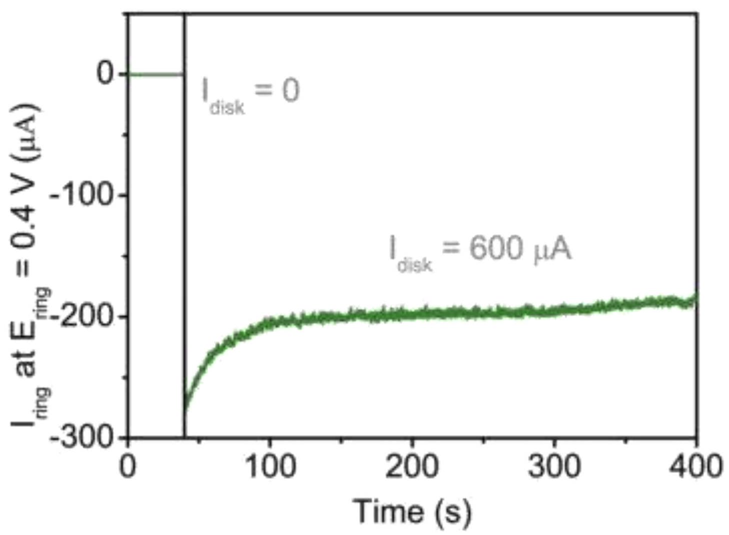

Supplementary Figure 14: RRDE testing for NiFe-MOF in 0.1 M KOH solution; the ring current on an RRDE (1500 rpm) in KOH solution (ring potential: 0.4 V *vs.* RHE)

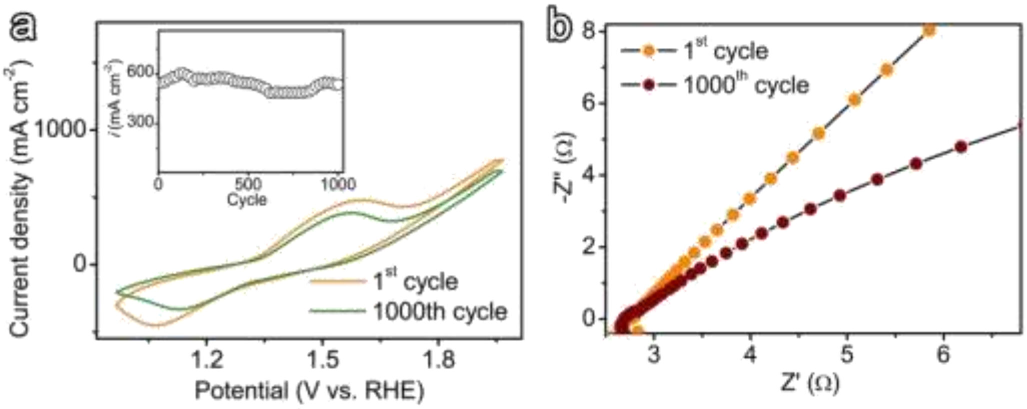

Supplementary Figure 15: Electrochemical data for NiFe-MOF during continuous potential cycling. (**a**) Cyclic voltammetry of NiFe-MOF before and after 1000 cycles at the scan rate of 50 mV s^{-1} from 0.966 to 1.966 V (*vs.* RHE) in 0.1 M KOH electrolyte; the inset of (**a**) shows the corresponding current density during cyclic voltammetry at 1.766 V (*vs.* RHE); (**b**) EIS spectra of NiFe-MOF before and after 1000 cycles.

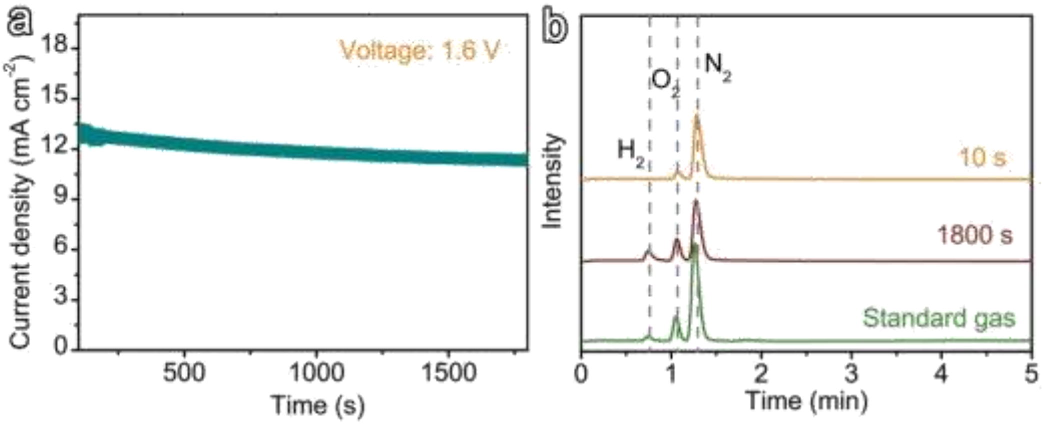

Supplementary Figure 16: Products analysis for NiFe-MOF electrode during water electrolysis in 0.1 M KOH electrolyte. (**a**) chronoamperometric response at 1.6 V for 1800 s; (**b**) Gas chromatography spectra of the product gas collected at the operation duration of 10 s and 1800 s, in comparison to the standard gas (H_2 1%, O_2 1%, CO 1%, CO_2 1%, CH_4 1%, N_2 95%). The experiment was performed in N_2 atmosphere, therefore a large peak for N_2 at around 1.3 min was observed. Also, the peak at around 1.1 min was identified for oxygen gas, while the peak at 0.8 min as the hydrogen gas. Other than N_2, O_2, and H_2, no other gases are detected by the gas chromatography instrument.

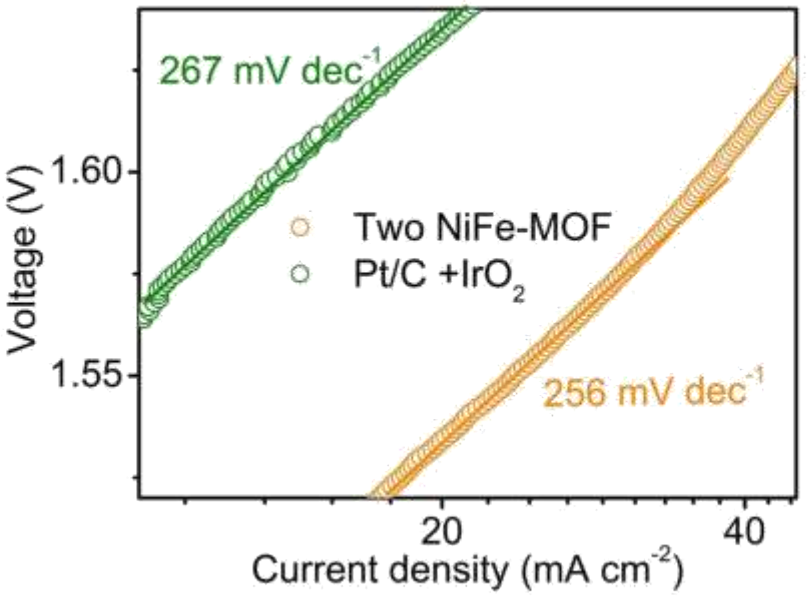

Supplementary Figure 17: Tafel plot of NiFe-MOF for overall water splitting, in comparison to Pt/C+IrO_2 benchmark. The smaller Tafel slope of NiFe-MOF (256 mV dec^{-1}) indicates its more favorable reaction kinetics than Pt/C+IrO_2 (267 mV dec^{-1}) .

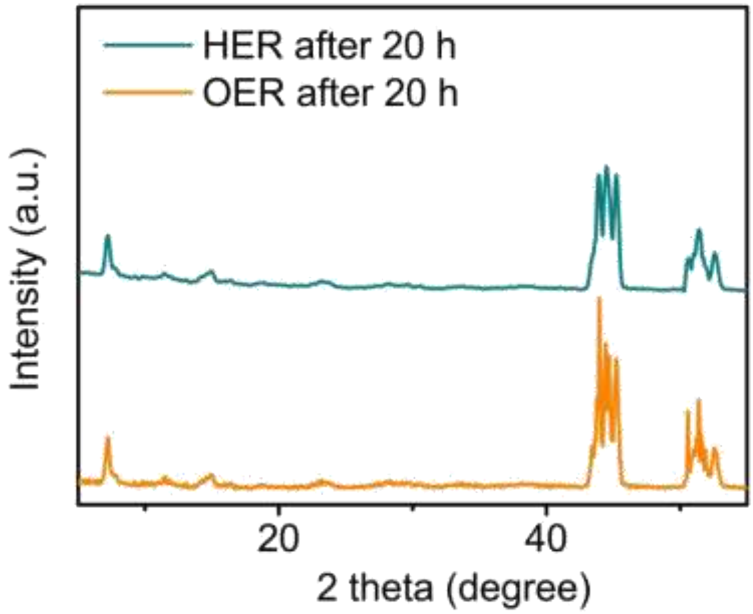

Supplementary Figure 18: XRD profiles of NiFe MOF electrode after 20 h bulk water electrolysis tests at a cell voltage of 1.5 V in 0.1 M KOH electrolyte.

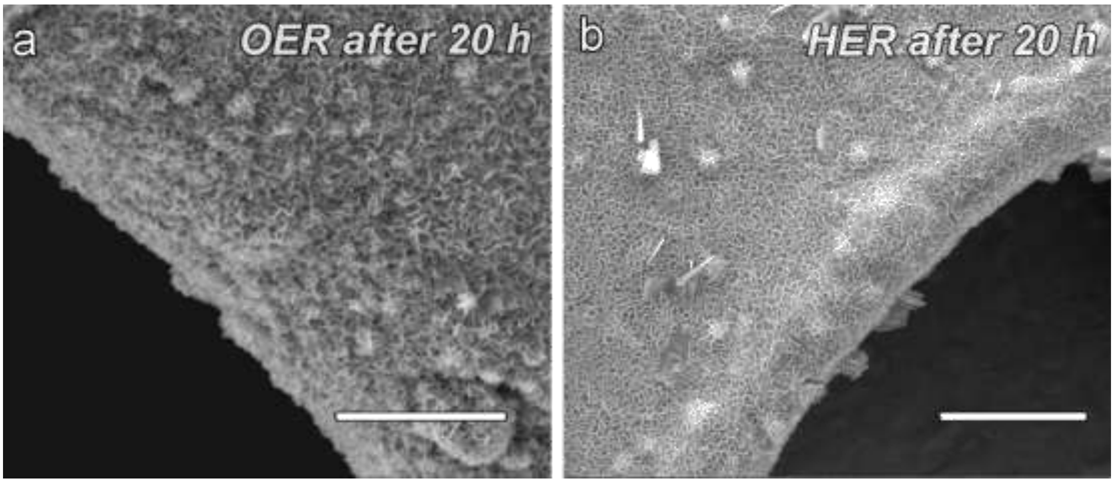

Supplementary Figure 19: SEM images of the NiFe-MOF electrode after 20 h bulk water electrolysis tests at a cell voltage of 1.5 V in 0.1 M KOH electrolyte. (**a**) OER anode; (**b**) HER cathode. Scale bar in **a** represents for 3 μm, and **b** for 10 μm.

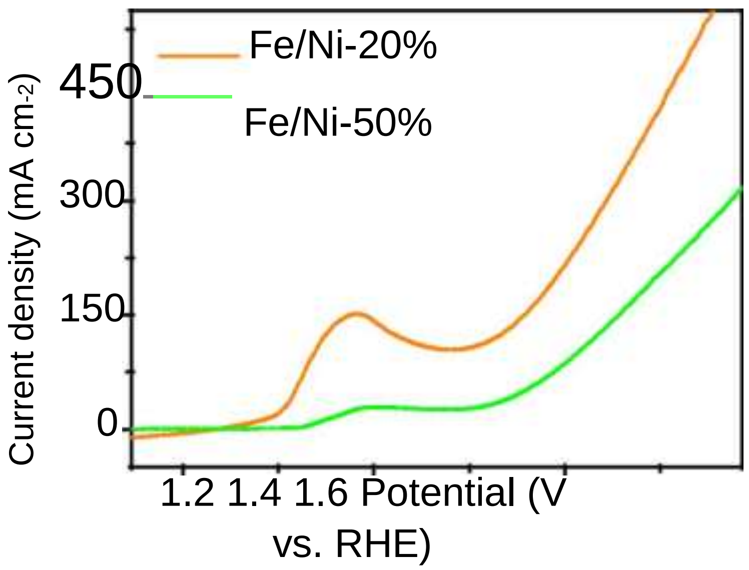

Supplementary Figure 20: LSV plots of NiFe-MOF with different Fe/Ni ratios at the scan rate of 10 mV s^{-1} in 0.1 M KOH electrolyte. It is shown that 0.25:1 of Fe/Ni in side NiFe-MOF has high catalytic current densities than that of 1:1 counterpart.

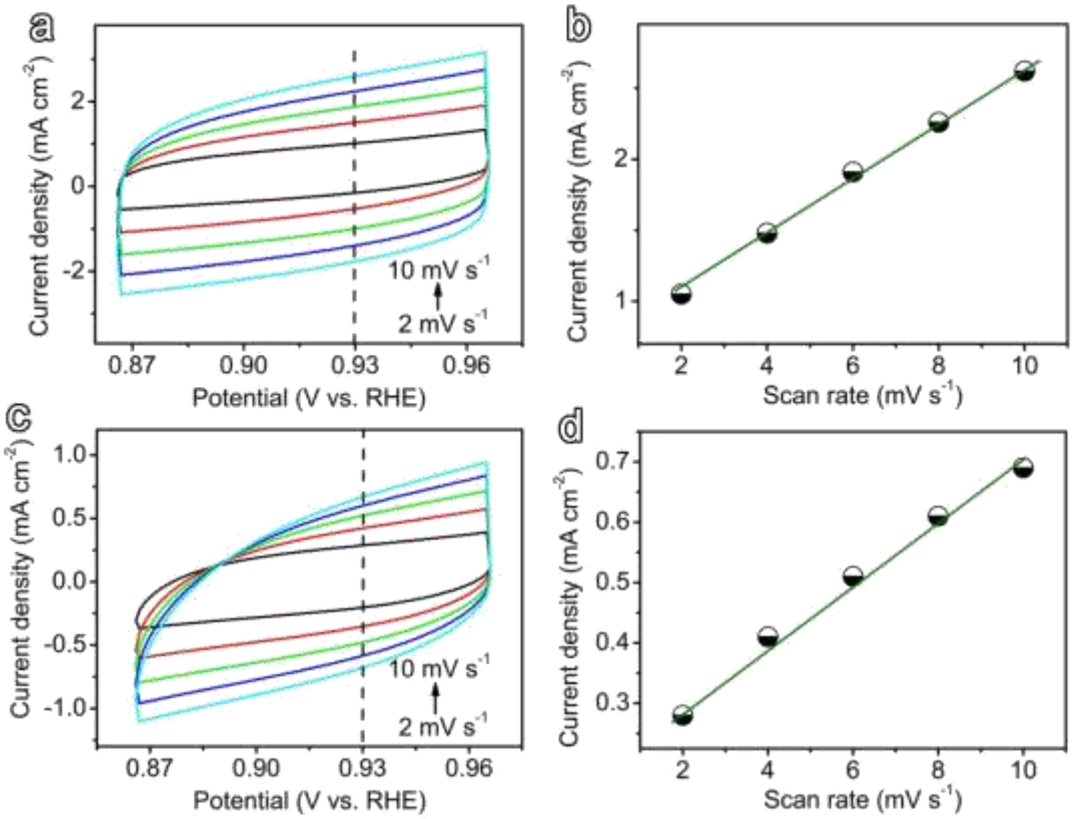

Supplementary Figure 21: Capacitance study of the prepared of different samples. (**a,b**) NiFe -MOF and (**c,d**) bulk NiFe- MOF; (**a,c**) the corresponding CVs measured at different scan rates from 2 to 10 mV s^{-1} in a potential region of 0.86 ~ 0.97 V (*vs.* RHE); (**b,d**) the current density at 0.93 V (*vs.* RHE) was plotted vs. scan rate. The C_{dl} value of the synthesized electrode was evaluated on the basis of CVs. The CVs of both samples exhibit a typical rectangular shape of an electrical double layer capacitor. In this potential region, the charge transfer electrode reactions are considered to be negligible and the current originates solely from electrical double layer charging and discharging. The plot of current density (at 0.93 V *vs.* RHE) against scan rate has a linear relationship, and its slope is the double layer capacitance.

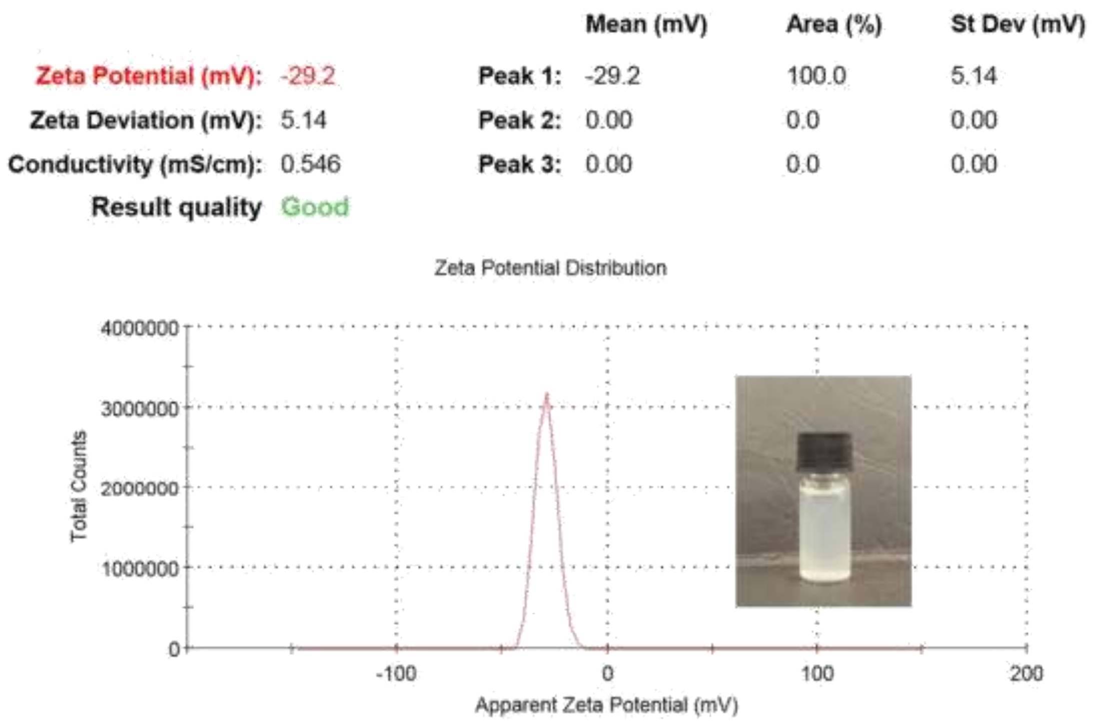

Supplementary Figure 22: The zeta potential of NiFe-MOF nanosheet in water. The testing dispersion was prepared by bath ultrasonication (10 min) of NiFe-MOF (1 cm^2) in de-ionized water (2 mL). The inset is a photograph of NiFe MOF-water dispersion.

Supplementary Note 3: The zeta potential of NiFe-MOF is -29.2 mV, indicating the sheet is negatively charged in water. Moreover, NiFe- MOF nanosheet can form homogeneous dispersion in water, as shown by the photograph (The vial is 1 cm in diameter).

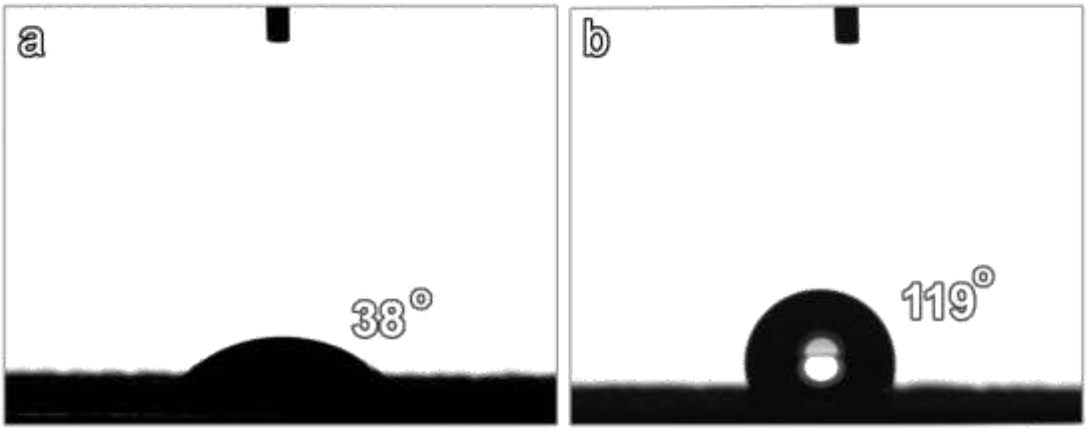

Supplementary Figure 23: Contact angle measurements of different samples. (**a**) NiFe-MOF (**b**) Calcined NiFe-MOF at 650 ^{o}C for 4 h. It is seen that NiFe-MOF is hydrophilic with the contact angle of only 38°, which is different from the calcined sample of 119 ^{o}C because of containing carbons.

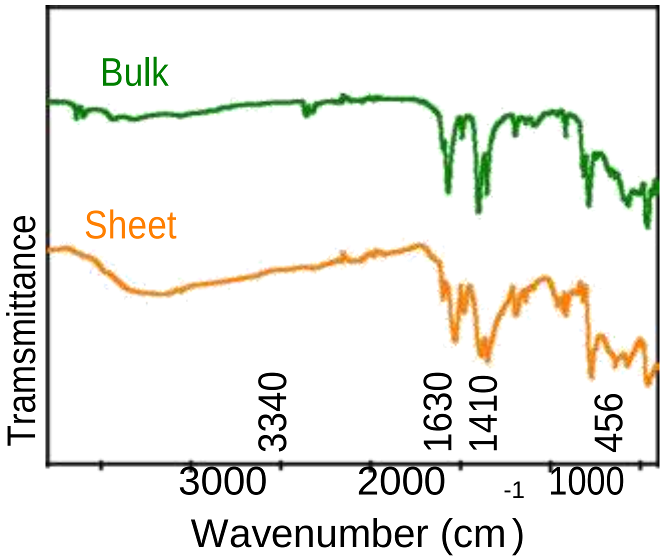

Supplementary Figure 24: FTIR of NiFe-MOF ultrathin nanosheet in comparison to its bulk counterpart. All the characteristic peaks are similar for both samples, indicating they have a similar metal-organic framework surface.

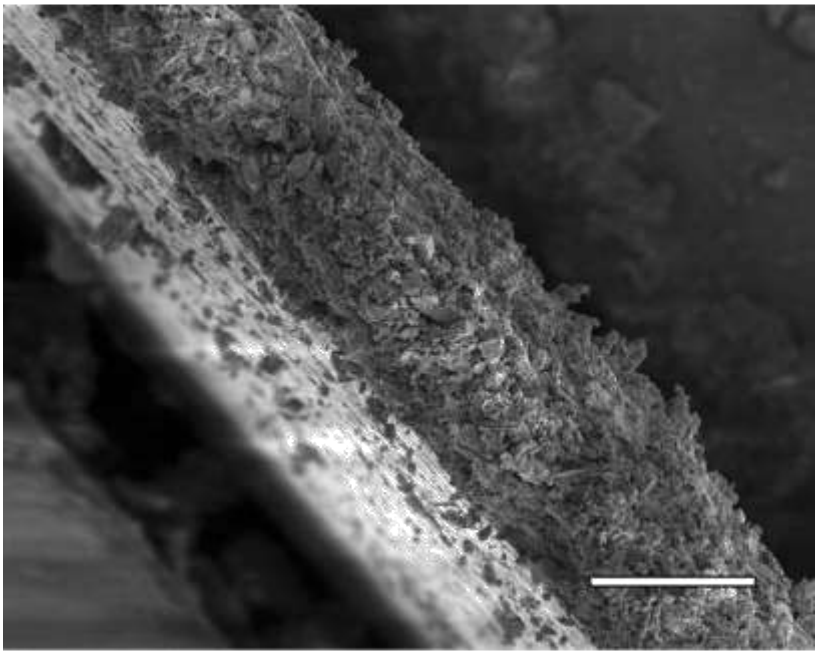

Supplementary Figure 25: SEM image of the NiFe MOF thin film (thickness ~ 240 nm) for four-point probe testing. The scale bar represents for 200 μm.

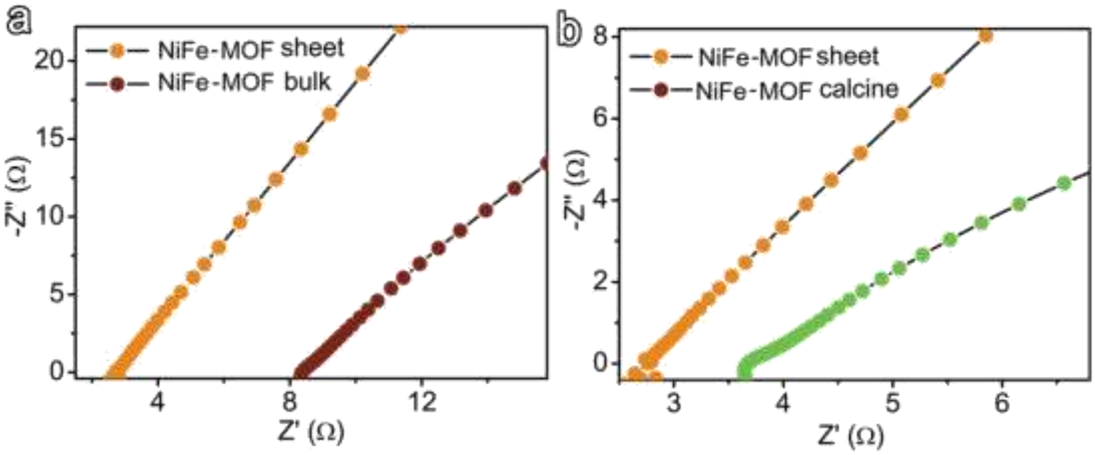

Supplementary Figure 26: EIS spectra of NiFe-MOF in comparison to its bulk and calcined counterparts in 0.1 M KOH electrolyte, showing that NiFe-MOF sheet have the lowest internal resistance of 2.8 ohm than other samples (8.2 ohm for bulk and 3.6 ohm for the calcined samples).

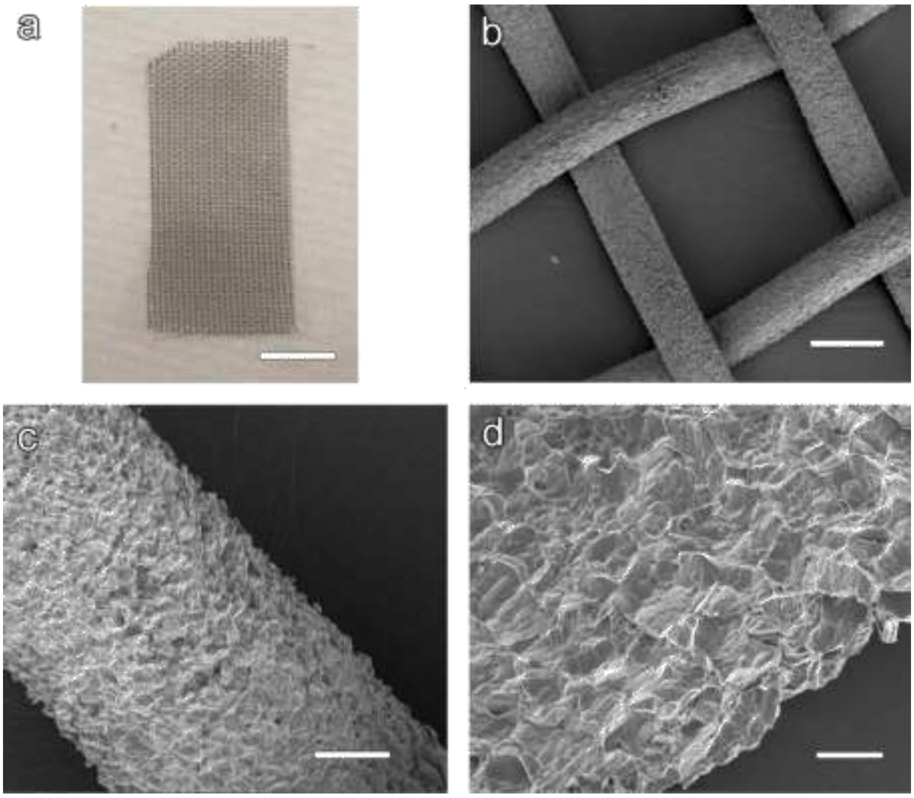

Supplementary Figure 27: Characterizations of NiFe-MOF grown on stainless steel mesh substrate. (**a**) the photograph; (**b-d**) SEM images of the material. The scales bar in **a-d** represent for 0.5 cm, 100, 20, and 5 µm**.**

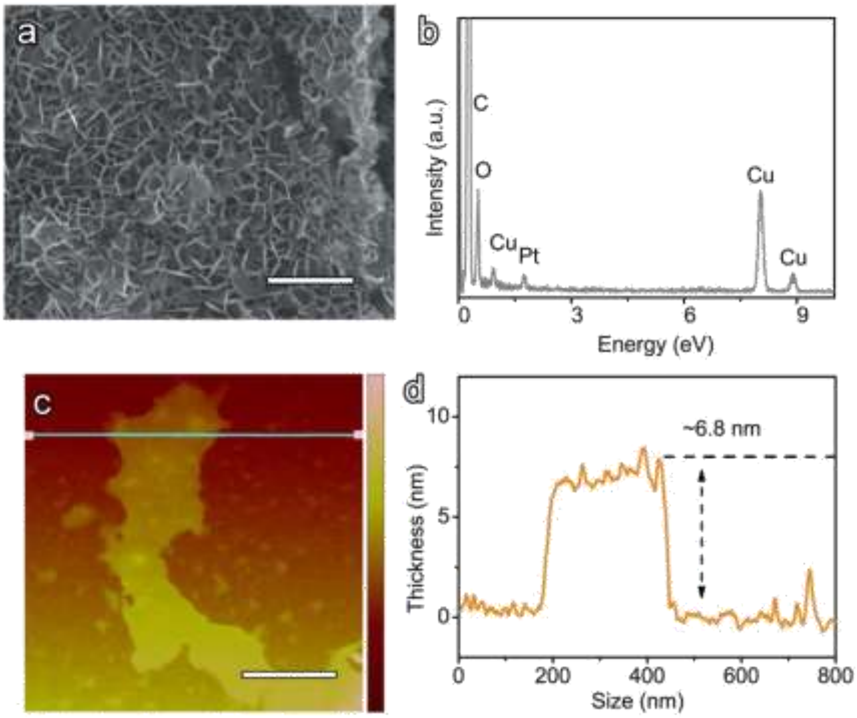

Supplementary Figure 28: Characterizations of Cu-MOF grown on nickel foam. (**a**) SEM image; (**b**) EDS spectrum; (**c,d**) AFM and corresponding height profile. The scale bars in **a** and **c** represent for 1 µm and 200 nm.

Supplementary Table 1. Comparison of the OER activity for the synthesized NiFe-MOF with several recently reported highly active electrocatalysts.

Catalyst	η_{10} (V)	Mass loading	Electrolyte	Supplementary Reference no.
NiFe-MOF	0.24	0.3 mg/cm^2	0.1 M KOH	This work
IrO_2	0.32	0.3 mg/cm^2	0.1 M KOH	This work
$Co^{II}_3Co^{III}_2(C_3H_3N_2)_{12}$ MOF	0.83	1 mg/cm^2	1 M NaOH	1
MOF-derived Co_3O_4/carbon nanowire arrays	0.29	0.2 mg/cm^2	0.1 M KOH	2
N- graphene/CoO	0.36	0.7 mg/cm^2	1 M KOH	3
Cobalt borate/graphene	0.29	0.285 mg/cm^2	1 M KOH	4
$Zn_xCo_{3-x}O_4$ nanowires arrays	0.32	1 mg/cm^2	1 M KOH	5
Rutile RuO_2	>0.47	0.05 mg/cm^2	0.1 M KOH	6
C_3N_4-CNT composite	0.37	0.2 mg/cm^2	0.1 M KOH	7
N-doped graphitic carbon	0.38	0.2 mg/cm^2	0.1 M KOH	8
NiFe ultrathin nanosheets	0.3	0.07 mg/cm^2	1 M KOH	9
Thin-film NiFe oxides	0.3	Not available	0.1 M KOH	10
Ti^{4+} doped NiFe LDH	0.26	Not available	1 M KOH	11
$LaCoO_3$	0.55	0.25 mg/cm^2	0.1 M KOH	12

Supplementary Table 2. Comparison of the HER activity for the synthesized NiFe-MOF with several recently reported highly active electrocatalysts.

Catalyst	η_{10} (V)	Mass loading	Electrolyte	Supplementary Reference no.
NiFe-MOF	0.134	0.3 mg/cm^2	0.1 M KOH	This work
NiFe LDH	0.21	Not available	1 M NaOH	13
Porous Co phosphide/Phosphate	0.38	0.1 mg/cm^2	1 M KOH	14
Ni_2S_3 nanosheet array	0.223	1.6 mg/cm^2	1 M NaOH	15
Co/CoO/N-doped carbon	0.235	0.42 mg/cm^2	1 M NaOH	16
MoS_2/Ni2S_3	0.11	9.7 mg/cm^2	1 M KOH	17
MC_x nano-octahedrons	0.15	0.8 mg/cm^2	1 M KOH	18
CoSe/NiFe LDH	0.26	4 mg/cm^2	1 M KOH	19
Cobalt-Embedded Nitrogen-Rich Carbon Nanotubes	0.37	0.28 mg/cm^2	1 M KOH	20

Supplementary Reference

1 Flugel, E. A., Lau, V. W., Schlomberg, H., Glaum, R. & Lotsch, B. V. Homonuclear mixed-Valent Cobalt Imidazolate Framework for Oxygen-Evolution Electrocatalysis. *Chem. Eur. J.* **22**, 3676-3680 (2016).

2 Ma, T. Y., Dai, S., Jaroniec, M. & Qiao, S. Z. Metal-organic framework derived hybrid Co_3O_4-carbon porous nanowire arrays as reversible oxygen evolution electrodes. *J. Am. Chem. Soc.* **136**, 13925-13931 (2014).

3 Mao, S., Wen, Z., Huang, T., Hou, Y. & Chen, J. High-performance bi-functional electrocatalysts of 3D crumpled graphene–cobalt oxide nanohybrids for oxygen reduction and evolution reactions. *Energy Environ. Sci.* **7**, 609-616 (2014).

4 Chen, P. *et al.* Strong-coupled cobalt borate nanosheets/graphene hybrid as electrocatalyst for water oxidation under both alkaline and neutral conditions. *Angew. Chem. Int. Ed.* **55**, 2488-2492 (2016).

5 Li, Y., Hasin, P. & Wu, Y. $Ni_xCo_{3-x}O_4$ nanowire arrays for electrocatalytic oxygen evolution. *Adv. Mater.* **22**, 1926-1929 (2010).

6 Lee, Y., Suntivich, J., May, K. J., Perry, E. E. & Shao-Horn, Y. Synthesis and Activities of Rutile IrO_2 and RuO_2 Nanoparticles for Oxygen Evolution in Acid and Alkaline Solutions. *J. Phys. Chem. Lett.* **3**, 399-404 (2012).

7 Ma, T. Y., Dai, S., Jaroniec, M. & Qiao, S. Z. Graphitic carbon nitride nanosheet–carbon nanotube three-dimensional porous cComposites as high-performance oxygen evolution electrocatalysts. *Angew. Chem. Int. Ed.* **53**, 7281-7285 (2014).

8 Zhao, Y., Nakamura, R., Kamiya, K., Nakanishi, S. & Hashimoto, K. Nitrogen-doped carbon nanomaterials as non-metal electrocatalysts for water oxidation. *Nat. Commun.* **4**, 2390 (2013).

9 Song, F. & Hu, X. Exfoliation of layered double hydroxides for enhanced oxygen evolution catalysis. *Nat. Commun.* **5**, 4477 (2014).

10 Louie, M. W. & Bell, A. T. An investigation of thin-film Ni–Fe oxide catalysts for the electrochemical evolution of oxygen. *J. Am. Chem. Soc.* **135**, 12329-12337 (2013).

11 Hunter, B. M. *et al.* Highly active mixed-metal nanosheet water oxidation catalysts made by pulsed-laser ablation in liquids. *J. Am. Chem. Soc.* **136**, 13118-13121 (2014).

12 Zhou, S. *et al.* Engineering electrocatalytic activity in nanosized perovskite cobaltite through surface spin-state transition. *Nat. Commun.* **7**, 11510 (2016).

13 Luo, J. *et al.* Water photolysis at 12.3% efficiency via perovskite photovoltaics and Earth-abundant catalysts. *Science* **345**, 1593-1596 (2014).

14 Yang, Y., Fei, H., Ruan, G. & Tour, J. M. Porous cobalt-based thin film as a bifunctional catalyst for hydrogen generation and oxygen generation. *Adv. Mater.* **27**, 3175-3180 (2015).

15 Feng, L. L. *et al.* High-index faceted Ni_3S_2 nanosheet arrays as highly active and ultrastable electrocatalysts for water splitting. *J. Am. Chem. Soc.* **137**, 14023-14026 (2015).

16 Jin, H. *et al.* In situ cobalt-cobalt oxide/N-doped carbon hybrids as superior bifunctional electrocatalysts for hydrogen and oxygen evolution. *J. Am. Chem. Soc.* **137**, 2688-2694 (2015).

17 Zhang, J. *et al.* Interface Engineering of MoS_2/Ni_3S_2 Heterostructures for Highly Enhanced Electrochemical Overall-Water-Splitting Activity. *Angew. Chem. Int. Ed.* **128**, 6814-6819 (2016).

18 Wu, H. B., Xia, B. Y., Yu, L., Yu, X.-Y. & Lou, X. W. D. Porous molybdenum carbide nano-octahedrons synthesized via confined carburization in metal-organic frameworks for efficient hydrogen production. *Nat. Commun.* **6** (2015).

19 Hou, Y. *et al.* Vertically oriented cobalt selenide/NiFe layered-double-hydroxide nanosheets supported on exfoliated graphene foil: an efficient 3D electrode for overall water splitting. *Energy Environ. Sci.* **9**, 478-483 (2016).

20 Zou, X. *et al.* Cobalt-embedded nitrogen-rich carbon nanotubes efficiently catalyze hydrogen evolution reaction at all pH values. *Angew. Chem. Int. Ed.* **126**, 4461-4465 (2014).
